# Editorial: Extinction Learning from a Mechanistic and Systems Perspective

**DOI:** 10.3389/fnbeh.2016.00115

**Published:** 2016-06-13

**Authors:** Denise Manahan-Vaughan, Oliver T. Wolf, Onur Güntürkün

**Affiliations:** ^1^Department of Neurophysiology, Medical Faculty, Ruhr University BochumBochum, Germany; ^2^Department of Cognitive Psychology, Faculty of Psychology, Institute of Cognitive Neuroscience, Ruhr University BochumBochum, Germany; ^3^Department of Biopsychology, Faculty of Psychology, Institute of Cognitive Neuroscience, Ruhr University BochumBochum, Germany

**Keywords:** extinction learning, fear conditioning, appetitive learning, predictive learning, Pavlovian conditioning, brain structure, neurotransmitter, renewal

Throughout life, we learn to associate stimuli with their consequences. But some of the new information that we encounter forces us to abandon what we had previously acquired. This old information is then subject to a new learning process that is called *extinction learning*. This involves a large number of brain structures (Kattoor et al., 2013; Lissek et al., 2013, Lissek et al.; Merz et al., 2014). Extinction is an unusually complex learning process that can involve both Pavlovian (classical; Pavlov, 1927; Lattal and Lattal, 2012) and operant (instrument) conditioning (Skinner, 1938; Bouton et al., 2012). A further hallmark is its context-dependency (Bouton, 2004) that is likely to rely on a tight interaction between the hippocampus and other brain areas (e.g., André et al.; Icenhour et al., 2015). Thus, one of the aims of the present Research Topic was to incorporate studies that analyze the concert of neural structures that enable extinction learning.

The old memory trace may be partly, or not at all forgotten during extinction (Üngör and Lachnit, 2006). It tends to re-emerge after a passage of time (spontaneous recovery), when re-exposure to the context of original learning occurs (renewal), or unexpected exposure to the unconditioned stimulus takes place (reinstatement). Such invasive memories are key symptoms of anxiety or pain disorders. They especially occur in individuals with enhanced susceptibility (Mosig et al.; Glombiewski et al., 2015). Although pathological fear in anxiety disorders can be treated through extinction-based approaches, treatment is not always successful in the long-term, underscoring the need to understand the mechanisms underlying impaired extinction. Therefore, the second aim of the Research Topic was to include publications that are situated at the transition between basic and clinical neuroscience.

Given the relevance of extinction, it is astonishing how little we know about extinction learning, in terms of its neural fundaments and its development, especially when moving outside the realm of fear extinction in rodents. The third aim of the Research Topic was therefore to include papers on the uncharted territories of extinction learning that involve less-studied entities such as the immune system (Hadamitzky et al., 2016) or hormonal factors (Wolf et al., 2015; Maren and Holmes, 2016), less-studied species (Lengersdorf et al.) or novel paradigms (Wiescholleck et al., 2014).

One specific goal of this Research Topic was to offer a basis for trans-species comparisons, as reflected by the spectrum of animals described that range from snails, through mice, rats, and pigeons. Several of the studies also describe extinction learning in humans, including pharmacological approaches. A number of studies (André et al.; Lengersdorf et al.; André and Manahan-Vaughan; Andrianov et al.; de Oliveira et al.; Lissek et al.) addressed neurotransmitter systems that are known to be involved in other forms of learning (Morris, 2013; Seyedabadi et al., 2014; Bauer, 2015) and in synaptic plasticity that is believed to underlie learning (Harley, 2004; Lesch and Waider, 2012; Park et al., 2013; Hansen and Manahan-Vaughan, 2014; Hagena et al., 2015). Here, for example, antagonism of N-methyl-D-aspartate receptors (NMDAR) prevented appetitive extinction in pigeons (Lengersdorf et al.), and GluN2B-containing NMDAR were found to play a key role in extinction of conditioned suppression of licking in rats (de Oliveira et al.). In an interesting corollary to the latter finding, Shumake and Monfils describe how conditioned suppression of licking is far more sensitive to extinction than freezing behavior, and along with Lee et al. investigated the impact of reactivating the original memory trace on extinction success. Examination of the role of dopamine receptors in appetitive learning in rats (André and Manahan-Vaughan) and predictive learning in humans (Lissek et al.), highlight differences that may relate to the species, or the extinction learning paradigm studied.

Studies with regard to the neural basis of extinction learning, and its associated brain structures, revealed a specific and experience-dependent role of microcircuitry within the basolateral amygdala (Sangha). In their review article, Giustino and Maren challenge the common assumption that the medial prefrontal cortex (mPFC) mediates the expression, whereas the infralimbic cortex (IL) mediates the suppression of fear responses, whereas Lee at al. offer experimental evidence that extinction learning and retrieval trigger differentiated responses in the mPFC and amygdala. Goodman and Packard differentiated between extinction learning of response and place learning, and provide evidence that the effectivity of the extinction learning strategy depends on the memory system (dorsolateral striatum vs. hippocampus) that encoded the original experience. In line with studies in rats (Gershman et al.), Shiban et al. observed that gradually reducing the frequency of aversive stimuli, in a Pavlovian fear conditioning paradigm in humans, is more effective in averting the return of fear than abrupt stimulus withdrawal, and Zlomuzica et al. demonstrate that improved self-efficacy also improves fear extinction. By contrast, Vervliet and Indekkeu show that low-cost avoidance behavior is resilient to extinction. Earlier studies indicate that extinction learning is reinforced by a context change (Bouton, 2004). Here, Sjouwerman et al. report that the timing of the context change is decisive with regard to the functional outcome with regard to both extinction and renewal. At the structural and/or molecular levels, several studies provided evidence for the direct involvement of the hippocampus in extinction learning (Lissek et al.; de Oliveira et al.; Wille et al.). Whereas, de Oliveira et al. provide evidence of the involvement of the dorsal hippocampus in conditioned suppression, Wille et al. describe how modulation of the expression of chromatin remodeling factors in the ventral hippocampus rescue impaired extinction of conditioned fear. Several studies examined hormonal control of extinction learning in fear, or stress-based, paradigms (Perez-Torres et al.; Hadad-Ophir et al.; Labrenz et al.): aspects that were also addressed in a review article by Stockhorst and Antov and a research perspective by Elsenbruch and Wolf.

What becomes apparent from these studies is the emergence of fine-tuning of our understanding as to which neural structures regulate extinction learning, what common denominators (and differences) exist between species, and how the regulation of extinction learning by neurotransmitter systems aligns with current knowledge as to the role of these systems in learning and memory. The papers compiled in this Research Topic offer new and valuable insights into the mechanisms and functional implementation of extinction learning at its different levels of complexity, and form the basis for new concepts and research ideas in this field.

## Author contributions

All authors listed, have made substantial, direct and intellectual contribution to the work, and approved it for publication.

### Conflict of interest statement

The authors declare that the research was conducted in the absence of any commercial or financial relationships that could be construed as a potential conflict of interest.
